# Proprotein Convertase Subtilisin/Kexin Type 9 Induction in COVID-19 Is Poorly Associated with Disease Severity and Cholesterol Levels

**DOI:** 10.3390/idr16040045

**Published:** 2024-07-17

**Authors:** Patricia Mester, Pablo Amend, Stephan Schmid, Jürgen J. Wenzel, Marcus Höring, Gerhard Liebisch, Sabrina Krautbauer, Martina Müller, Christa Buechler, Vlad Pavel

**Affiliations:** 1 Department of Internal Medicine I, Gastroenterology, Hepatology, Endocrinology, Rheumatology and Infectious Diseases, University Hospital Regensburg, 93053 Regensburg, Germany; patricia.mester@klinik.uni-regensburg.de (P.M.); pablo.amend@stud.uni-regensburg.de (P.A.); stephan.schmid@klinik.uni-regensburg.de (S.S.); martina.mueller-schilling@klinik.uni-regensburg.de (M.M.); vlad.pavel@klinik.uni-regensburg.de (V.P.); 2 Institute of Clinical Microbiology and Hygiene, University Hospital Regensburg, 93053 Regensburg, Germany; juergen.wenzel@klinik.uni-regensburg.de; 3 Institute of Clinical Chemistry and Laboratory Medicine, University Hospital Regensburg, 93053 Regensburg, Germany; marcus.hoering@klinik.uni-regensburg.de (M.H.); gerhard.liebisch@klinik.uni-regensburg.de (G.L.); sabrina.krautbauer@klinik.uni-regensburg.de (S.K.)

**Keywords:** COVID-19, intensive care, mortality, free cholesterol, cholesteryl ester, bacterial infection

## Abstract

SARS-CoV-2 infection was shown to induce proprotein convertase subtilisin/kexin type 9 (PCSK9) plasma levels in sepsis. Here, we investigate the association between serum PCSK9 levels and disease severity. PCSK9 was measured in serum of 55 controls, 40 patients with moderate and 60 patients with severe COVID-19 disease. Serum PCSK9 was elevated in moderate COVID-19 compared to controls and further increased in severe cases. PCSK9 levels were not associated with C-reactive protein, bacterial superinfections, interventions, or survival in patients with severe COVID-19. PCSK9 regulates circulating cholesterol levels, and 15 cholesteryl ester (CE) species and free cholesterol (FC) were quantified by direct flow injection analysis using a high-resolution hybrid quadrupole-Orbitrap mass spectrometer. Most CE species with shorter fatty acid chains were decreased in severe compared to moderate COVID-19, and none of the CE species were correlated with PCSK9 in patients with severe COVID-19. Levels of all CE species negatively correlated with C-reactive protein in severe COVID-19 patients. Notably, FC was induced in severe compared to moderate COVID-19. The FC/CE ratio correlated positively with inflammatory markers and was associated with non-survival. The current study suggests that the imbalance between CE and FC levels is associated with disease severity and mortality in patients with COVID-19.

## 1. Introduction

Severe acute respiratory syndrome coronavirus type 2 (SARS-CoV-2) is the causative agent of coronavirus disease-2019 (COVID-19). The symptoms of COVID-19 vary greatly, ranging from asymptomatic to severe cases requiring intensive care treatment [1]. Among the most severe complications of COVID-19, acute respiratory distress syndrome (ARDS) stands out with a high mortality rate [2,3,4]. Individuals with advanced age, obesity, and metabolic comorbidities such as type 2 diabetes are at an increased risk of developing severe forms of COVID-19 [5]. Low-grade chronic inflammation and impaired immunity are consistently related to obesity and metabolic diseases and are suggested to contribute to COVID-19 disease severity [6].

Proprotein convertase subtilisin/kexin type 9 (PCSK9) targets the low-density lipoprotein (LDL) receptor for degradation and increases serum LDL levels (Figure S1). Serum PCSK9 levels of obese subjects are higher in comparison to normal-weight controls and may contribute to dyslipidemia associated with overweight, obesity, and type 2 diabetes [6,7,8].

Anti-PCSK9 monoclonal antibodies, which block the binding of PCSK9 to the LDL receptor, have been developed. These inhibitory antibodies effectively lower LDL cholesterol levels [7].

Infectious diseases are known to be linked with reduced levels of LDL, high-density lipoprotein (HDL), and total blood cholesterol. The severity of the underlying diseases is negatively correlated with blood cholesterol levels. In patients with sepsis, total cholesterol, HDL-cholesterol, and apolipoprotein A-I (ApoA-I), the major apolipoprotein associated with HDL, are predictive of mortality [9,10]. During recovery from severe illness, circulating lipoprotein and serum cholesterol levels generally normalize [11]. As humans injected with lipopolysaccharide experience a strong decline in circulating lipids, this suggests that inflammation plays a causal role in lipid lowering in bacterial infections [12]. The mechanisms behind low cholesterol levels in severe illness are not yet fully understood, and there are conflicting data on the role of PCSK9 in this process [11].

Evidence suggests that PCSK9 inhibition may protect against systemic inflammation and sepsis [13]. Patients with PCSK9 loss-of-function mutations displayed lower interleukin (IL)-6 levels in the blood after lipopolysaccharide injection and had increased survival after sepsis [14]. PCSK9 gain-of-function mutation carriers had reduced survival in sepsis [15]. However, the use of PCSK9 inhibitors did not protect patients from severe illness. A recent meta-analysis of 20 randomized, double-blind, placebo-controlled trials concluded that treatment with PCSK9 blocking antibody did not increase the risk for sepsis, nor was this related to severe bacterial and viral infections [16]. Unexpectedly, PCSK9 blockage had a protective effect on SARS-CoV-2 infection. In a study on COVID-19 patients, a single injection of the monoclonal PCSK9 antibody evolocumab led to reduced serum IL-6 levels and improved outcomes compared to placebo at 30 days from baseline [17]. Notably, serum PCSK9 levels were not assessed before or during this study [17].

Plasma PCSK9 levels of septic patients due to COVID-19 were significantly higher in contrast to septic patients due to other causes. Plasma PCSK9 levels of the COVID-19 patients did not correlate with C-reactive protein (CRP), procalcitonin, IL-6, ferritin, or leukocyte count, illustrating that, at least in SARS-CoV-2 infection, higher PCSK9 is not simply a marker of inflammation [18].

While evidence suggests PCSK9’s role in COVID-19 severity, only one study has compared plasma PCSK9 levels between non-infected controls and SARS-CoV-2 infected patients, revealing higher levels in the latter [18]. Ruscica et al. reported PCSK9 levels in COVID-19 patients but lacked data on healthy controls [19]. Furthermore, to the best of our knowledge, no study has analyzed the serum levels of PCSK9 and cholesterol in COVID-19 patients simultaneously.

This study aims to investigate associations of serum PCSK9, serum cholesteryl ester species, and free cholesterol levels with COVID-19 severity in patients with moderate and severe COVID-19.

## 2. Materials and Methods

### 2.1. Study Cohort

Blood samples of the patients were collected between 16 April 2020, and 14 June 2021, during the hospital stay of the patients, who were aged 18 years or older. SARS-CoV-2 infection of all patients was confirmed by polymerase chain reaction (PCR). Blood samples were mostly collected within 72 h of hospital admission. In some patients, blood samples were taken a few days later due to delays in obtaining patient consent. Forty patients had tachycardia, pyrexia, difficulties in breathing, and weakness. These patients had systemic inflammatory response syndrome (SIRS) and were assigned to the group with moderate disease [20,21]. These patients were equivalent to moderate COVID-19, conforming to the National Institutes of Health (NIH) classification [22]. The patients were hospitalized but did not need intensive care. Sixty patients had to enter intensive care because they had ARDS and septic shock. The group of patients with severe COVID-19 illness corresponds to the category of critical illness as defined by the NIH classification [21,22,23,24]. The treatment of patients with COVID-19 was conducted in accordance with the current guidelines approved by the European Medicines Agency and the German Federal Joint Committee. In Germany, the drugs approved for the treatment of COVID-19 were remdesivir and dexamethasone. For thrombosis prophylaxis, all patients were administered low-molecular-weight heparin or unfractionated heparin. The vaccination against SARS-CoV-2 commenced in Germany on 26 December 2020. It appeared that the majority of our patients had not yet been vaccinated. At the time, drugs such as baricitinib, tocilizumab, or sotrovimab were not approved in Germany. Notably, this cohort does not overlap with our recent analysis [18].

The 55 healthy controls exhibited similar age distributions (58 (21–80) years) and sex ratios (26 females, 29 males) to those observed in patients with moderate and severe COVID-19.

### 2.2. ELISA

The DuoSet ELISA (R&D Systems; Wiesbaden, Nordenstadt, Germany) for analysis of human PCSK9 was used as indicated by the company. For analysis, serum was diluted 1:100-fold. All samples and standards were analyzed in duplicate with the mean values being employed for the purposes of calculations.

### 2.3. Measurement of Free Cholesterol and Cholesteryl Ester Levels

Non-naturally occurring internal standards were added before lipid extraction for quantitative lipidomics. A total of 10 µL serum was used for lipid extraction according to the protocol by Bligh and Dyer [25]. The analysis of cholesteryl ester (CE) and free cholesterol (FC) was performed by direct flow injection analysis (FIA) using a high-resolution hybrid quadrupole-Orbitrap mass spectrometer (FIA-FTMS). Recently, the FIA-FTMS method was described in detail [26,27]. CEs were recorded in positive ion mode m/z 500–1000 as [M+NH_4_]^+^ at a target resolution of 140,000 (at 200 m/z). CE species were corrected for their species–specific response. Analysis of FC was performed by multiplexed acquisition (MSX) of the [M+NH_4_]^+^ of FC and the deuterated internal standard (FC[D7]) [28].

### 2.4. Microbiological Tests

Microbiological analyses were conducted at the Institute of Clinical Microbiology and Hygiene (University Hospital Regensburg).

SARS-CoV-2-specific RNA was detected in respiratory specimens by reverse-transcription real-time quantitative PCR. An in-house assay with absolute quantification of target RNA was performed as previously described [29]. Quantitative results were expressed as genome copies per milliliter sample (cp/mL).

SARS-CoV-2 antibody levels were measured in serum by an ELISA detecting IgG antibodies against the SARS-CoV-2 receptor-binding domain as described previously [30].

Gram staining and blood culture were conducted for the diagnosis of bacterial blood infections. Microbial identification was performed by matrix-assisted laser desorption/ionization time-of-flight (MALDI-TOF) mass spectrometry (Bruker Microflex LT; Bruker, Hamburg, Germany) [31]. Resistance to vancomycin was identified by PCR analysis of the genes van A and van B. Herpes simplex virus was detected using PCR.

### 2.5. Routine Laboratory Testing

C-reactive protein (CRP) levels in serum were quantified by an enhanced method for immunoturbidimetric assays. IL-6 and procalcitonin levels were determined by the use of ElektroChemiLumineszenz ImmunoAssays. Lactate dehydrogenase (LDH) converts L-lactate to pyruvate, thereby producing NADH, which is determined by photometric measurement. The oxidation of L-lactate by lactate oxidase generates pyruvate and hydrogen peroxide, with the latter being detected colorimetrically. The activity of alkaline phosphatase (AP) is analyzed by the hydrolysis of p-nitrophenyl phosphate into phosphate and p-nitrophenol, which are measurable through photometry. Ferritin levels are determined by an ElektroChemiLumineszenz ImmunoAssay that uses ferritin-specific antibodies conjugated with ruthenium or biotin. The Cobas Pro analyzer and the corresponding assays from Roche (Penzberg, Germany) were used to measure the parameters described above. These tests were done at the Institute of Clinical Chemistry and Laboratory Medicine at the University Hospital Regensburg.

### 2.6. Statistical Analysis

Boxplots show the minimum, maximum, median, and quartiles. Outliers are shown as asterisks (extreme) or circles (mild). Tables present median, minimum, and maximum values. Statistical tests were the non-parametric tests Mann–Whitney-U, Kruskal–Wallis, and Spearman’s correlation, with categorical variables compared using the chi-square test (IBM SPSS Statistics 26.0 program). Receiver operating characteristic curve analysis was performed in SPSS (IBM, Chicago, IL, USA). Significance was defined as *p* < 0.05.

## 3. Results

### 3.1. Serum PCSK9 Levels of Healthy Controls and COVID-19 Patients

Here, we analyzed PCSK9 in the serum of 55 controls, 40 patients with moderate and 60 patients with severe COVID-19. Patients with severe COVID-19 disease exhibited elevated levels of CRP, procalcitonin, ferritin, and LDH in comparison to patients with moderate disease (Table 1). There was no significant difference in gender distribution, age, AP, IL-6, and lactate between the two groups (Table 1). The body mass index (BMI) of severe COVID-19 patients was higher in comparison to those with moderate disease. The viral load of patients with moderate and severe COVID-19 was similar, but the latter group had a higher antibody titer (Table 1).

**Figure 1:**
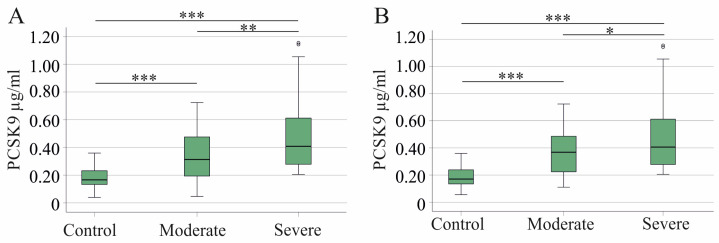
Serum PCSK9 levels of controls, moderate, and severe COVID-19 patients. (**A**) Serum PCSK9 of controls, patients with moderate and severe COVID-19; (**B**) Serum PCSK9 of controls, patients with moderate and severe COVID-19 after exclusion of COVID-19 patients with liver cirrhosis. * *p* < 0.05, ** *p* < 0.01, *** *p* < 0.001.

Serum PCSK9 was elevated in moderate COVID-19 in comparison to controls and was further increased in severe COVID-19 (Figure 1A). Four patients in the moderate and one in the severe group had liver cirrhosis, which was related to reduced PCSK9 in accordance with recent findings [18]. Exclusion of patients with liver cirrhosis caused a less significant difference in PCSK9 levels between patients with moderate and severe disease (Figure 1B).

PCSK9 serum levels of controls were 0.17 (0.06–0.36) µg/mL, of patients with moderate COVID-19 were 0.37 (0.11–0.72) µg/mL, and of patients with severe COVID-19 were 0.41 (0.20–1.16) µg/mL when patients with liver cirrhosis were excluded.

PCSK9 was suggested to reduce SARS-CoV-2 infection [32]. In the serum of patients with moderate COVID-19, PCSK9 positively correlated with viral load (r = 0.422, *p* = 0.012). Antibody levels were only known for 7 patients (Table 1), and data were not suitable for correlation analysis. In severe COVID-19 serum, PSCK9 was not correlated with viral titer (r = 0.102, *p* = 0.495) or antibody levels (r = −0.030, *p* = 0.842).

### 3.2. PCSK9 in Relation to Dialysis, Vasopressor Therapy, and Inflammation Markers

Serum PCSK9 was not related to the need for dialysis in the moderate (3 patients, *p* = 0.872) and severe (7 patients, *p* = 0.271) COVID-19 patients. The 41 patients with severe disease on vasopressor therapy had similar PCSK9 levels compared to patients without these drugs (*p* = 0.307).

In patients with severe COVID-19 disease, PCSK9 did not correlate with CRP, procalcitonin, ferritin, IL-6, LDH, AP, or lactate (*p* > 0.05 for all). Serum PCSK9 showed a positive correlation with CRP (r = 0.466, *p* = 0.004) and LDH (r = 0.370, *p* = 0.026) in the moderate group.

### 3.3. Effect of Bacterial and Fungal Superinfections and Herpes Simplex Virus Reactivation on Serum PCSK9 Levels

In patients with moderate COVID-19 disease, 4 patients were infected with bacteria, but this was not related to higher PCSK9 (*p* = 0.421). One patient had a fungal infection, and no patients had a reactivation of herpes simplex virus (HSV) or vancomycin-resistant enterococcus (VRE) superinfection.

In patients with severe disease, 27 patients had bacterial blood infections. PCSK9 levels did not vary between non-infected and infected patients (*p* = 0.712) and did not change in the 10 patients with VRE (*p* = 0.445). Fungal infections of 21 patients were not related to a change in PCSK9 (*p* = 0.226). It should be noted that patients with severe disease had a much higher rate of infection with bacteria (*p* < 0.001) and fungi (*p* < 0.001) compared to patients with moderate symptoms.

Reactivation of HSV is associated with COVID-19 [33] and occurred in 20 of our severe patients. These patients had similar PCSK9 (*p* = 0.955) as patients without HSV.

### 3.4. Serum PCSK9 and Survival

**Figure 2:**
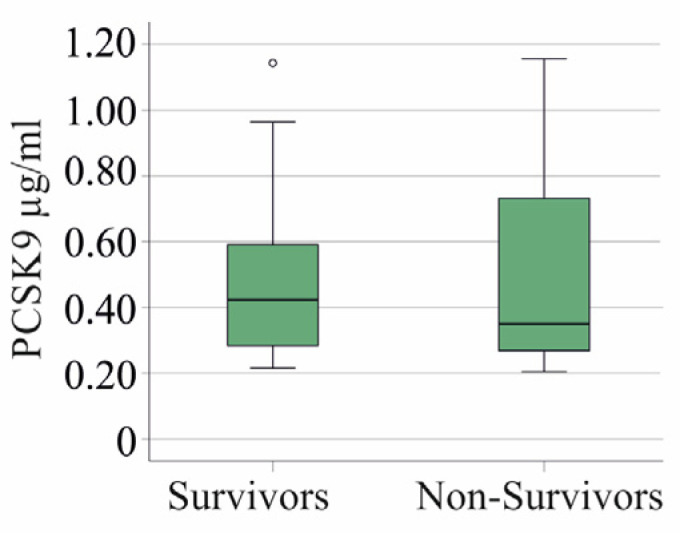
PCSK9 in the serum of patients with severe COVID-19 who survived and those who did not survive.

In the group of patients with severe COVID-19, 21 patients died because of the viral infection. In this group, PCSK9 levels between survivors and non-survivors did not differ (*p* = 0.585) (Figure 2).

### 3.5. Serum PCSK9 and Cholesterol Levels

PCSK9 is a protein that regulates serum cholesterol levels (Figure S1). High levels of PCSK9 are associated with elevated cholesterol [7]. Free cholesterol (FC) and 15 cholesteryl ester (CE) species were measured in the serum of our patients.

Patients with liver cirrhosis have low serum cholesterol [34] and in our entire cohort CE 18:1 (*p* = 0.047), CE 18:2 (*p* = 0.015), CE 18:3 (*p* = 0.010), CE 20:4 (*p* = 0.007), CE 20:5 (*p* = 0.009), and CE 22:6 (*p* = 0.002) and total cholesterol levels (*p* = 0.038) were reduced in cirrhosis, and these 5 patients were excluded.

Serum PCSK9 positively correlated with CE 18:3 (r = 0.351, *p* = 0.036) and CE 20:5 (r = 0.360, *p* = 0.031) in patients with moderate disease. PCSK9 did not correlate with any of the CE species, total CE levels, or FC in severe COVID-19 (*p* > 0.05 for all).

### 3.6. Serum Cholesterol and COVID-19 Severity

**Figure 3:**
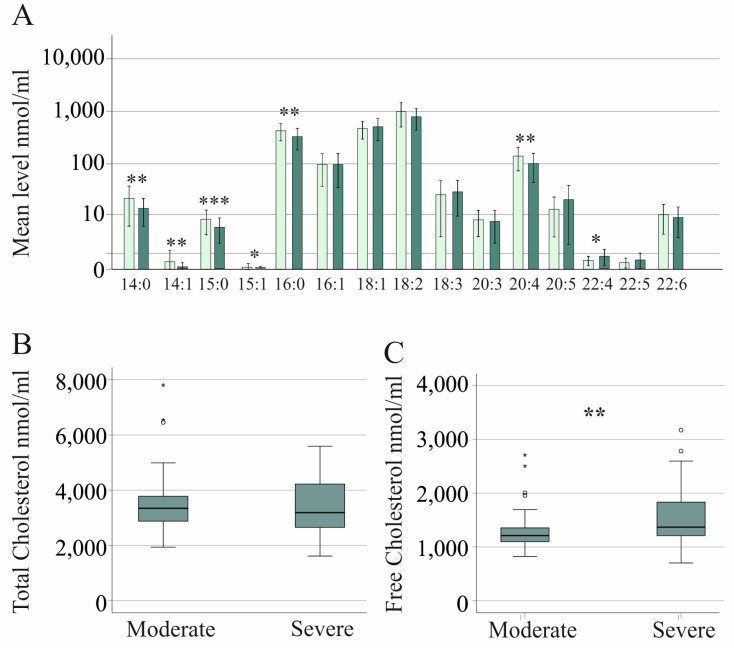
Serum cholesteryl ester and free cholesterol levels of COVID-19 patients. (**A**) Cholesteryl ester species of patients with moderate (light green bars) and severe (dark green bars) COVID-19; (**B**) Serum cholesterol levels (sum of all cholesteryl ester species and free cholesterol) of patients with moderate and severe COVID-19; (**C**) Serum free cholesterol levels of patients with moderate and severe COVID-19. * *p* < 0.05, ** *p* < 0.01, *** *p* < 0.001.

Patients with sepsis have low serum cholesterol levels, which are related to higher mortality [11,35]. CE species CE 14:0, 14:1, 15:0, 15:1, 16:0, and 20:4 were reduced in severe COVID-19, and CE 22:4 was increased in comparison to moderate cases (Figure 3A). Total cholesterol level, which is the sum of all CE species and FC, did not significantly change (Figure 3B). FC levels were increased in severe compared to moderate COVID-19 patients (Figure 3C).

**Figure 4:**
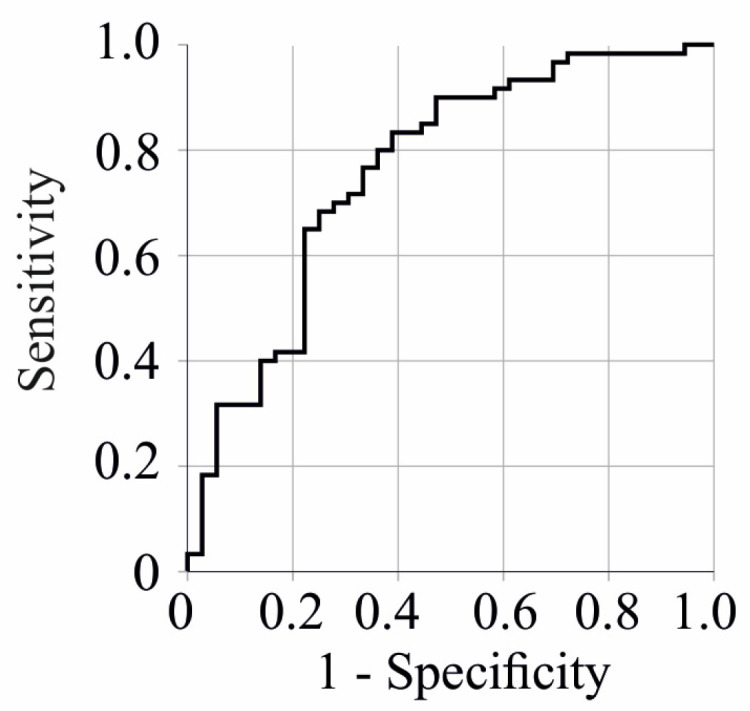
Receiver operating characteristic curve for free cholesterol/cholesteryl ester ratio to discriminate moderate and severe COVID-19 patients.

The areas under the receiver operating characteristic curve (AUROC) of CE species and FC for differentiation of severe from moderate COVID-19 patients were below 0.700, indicating poor discriminative power. The AUROC of the FC/CE ratio was 0.761 (*p* < 0.001) for the differentiation of severe from moderate COVID-19 patients (Figure 4). An FC/CE ratio of 0.6 had a sensitivity of 0.83 and a specificity of 0.61 for the diagnosis of severe COVID-19.

In severe COVID-19 CE 14:0 (r = −0.339, *p* = 0.018), CE 15:0 (r = −0.303, *p* = 0.036), CE 16:0 (r = −0.323, *p* = 0.025), and CE 20:4 (r = −0.382, *p* = 0.007) were negatively correlated with SARS-CoV-2 antibody levels. No associations were observed in the moderate group (*p* > 0.05 for all; please note that antibody titer was only documented for 7 patients). FC and CE species did not correlate with viral load in both cohorts (*p* > 0.05 for all). Viral load was negatively associated with antibody titer in severe COVID-19 (r = −0.334, *p* = 0.033).

### 3.7. Correlation of Cholesteryl Ester Species and Free Cholesterol Levels with Inflammation Markers, Superinfections, and Day of Blood Collection

In patients with moderate or severe COVID-19 disease, CE species and FC were not correlated with LDH, AP, or lactate (*p* > 0.05 for all). In severe COVID-19 patients, all CE species negatively correlated with CRP, 13 CE species with procalcitonin, 9 with IL-6, and 10 with ferritin. In the moderate group, one CE species correlated negatively with CRP and two with procalcitonin (Table 2). The FC/CE ratio was positively associated with all of these inflammatory markers in both cohorts (Table 2).

**Figure 5:**
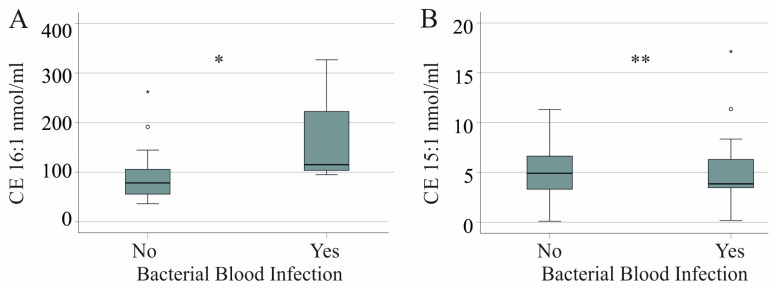
Cholesteryl ester (CE) and bacterial blood superinfection. (**A**) CE 16:1 level in patients with moderate COVID-19 without (No) and with (Yes) bacterial infection; (**B**) CE 15:1 level in patients with severe COVID-19 without (No) and with (Yes) bacterial infection. * *p* < 0.05. ** *p* < 0.01.

CE 16:1 (Figure 5A) and CE 22:4 (*p* = 0.035) were elevated in patients with moderate disease and bacterial infection. However, CE 15:1 was reduced in patients with severe COVID-19 and bacterial infections (Figure 5B). VRE and fungal infections were not related to a change in CE species levels, FC levels, and FC/CE ratio in severe COVID-19 (*p* > 0.05 for all). Reactivation of HSV was associated with a higher FC/CE ratio (*p* = 0.040).

Serum of patients with moderate COVID-19 was obtained 3 (1–16) days, and of patients with severe disease 4 (1–10) days after hospital admission. Serum PCSK9 did not correlate with the day of blood collection in the moderate (r = −0.004, *p* = 0.984) and severe (r = −0.029, *p* = 0.830) group. In the moderate and severe cohort, serum CRP, procalcitonin, and lactate dehydrogenase did not correlate with the day of blood collection, excluding that these clinical markers of disease severity are related to the day of blood collection.

In severe disease, day of blood collection positively correlated with CE 15:0 (r = 0.329, *p* = 0.010), CE 16:1 (r = 0.297, *p* = 0.021), CE 18:1 (r = 0.366, *p* = 0.004), CE 18:3 (r = 0.418, *p* = 0.001), CE 20:3 (r = 0.303, *p* = 0.019), CE 20:5 (r = 0.287, *p* = 0.026), total CE levels (r = 0.259, *p* = 0.045), and FC (r = 0.446, *p* < 0.001). In moderate disease, there was a negative correlation of CE 22:4 with day of blood collection (r = −0.358, *p* = 0.032).

### 3.8. PCSK9, Cholesteryl Ester Species and Free Cholesterol in Relation to Age, Sex, Body Mass Index, and Comorbidities

Serum levels of PCSK9, CE species, FC, and the FC/CE ratio between sexes of the moderate COVID-19 and the severe COVID-19 cohort were similar. PCSK9 did not correlate with the patient’s age in the moderate COVID-19 group (r = 0.242, *p* = 0.161) and the severe COVID-19 cohort (r = −0.093, *p* = 0.485). PCSK9 did not correlate with the BMI in the moderate COVID-19 group (r = −0.084, *p* = 0.732) and in the severe COVID-19 cohort (r = −0.027, *p* = 0.847).

CE 16:0 (r = −0.310, *p* = 0.018), CE 18:2 (r = −0.317, *p* = 0.015), CE 20:4 (r = −0.357, *p* = 0.006), CE 22:6 (r = −0.370, *p* = 0.004), and the FC/CE ratio (r = 0.383, *p* = 0.003) were correlated with age in the severe COVID-19 patients, whereas associations with age did not exist in the moderate cohort. In the moderate group, CE 20:3 was positively related with BMI, and in the severe cohort, 10 of the 15 analyzed CE species positively correlated with BMI (Table 3).

The number of hypertensive and type 2 diabetic patients did not differ between the moderate and severe COVID-19 cohorts. More patients were suffering from cardiovascular disease in the moderate group (Table 1).

Diabetes, cardiovascular disease, and hypertension in the moderate COVID-19 group were not associated with a change in serum PCSK9 or CE species levels. CE 16:0 (*p* = 0.020), CE 18:1 (*p* = 0.029), and CE 20:4 (*p* = 0.003) of patients with severe COVID-19 and diabetes were higher, and this may be in part associated with the increased BMI of diabetic patients (*p* = 0.011).

### 3.9. Cholesteryl Ester Species, Free Cholesterol Levels, and Survival

**Figure 6:**
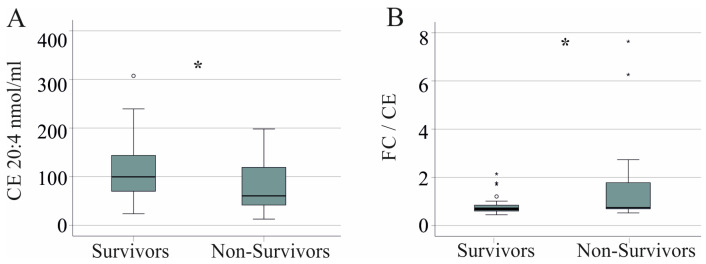
Cholesterol levels of survivors and non-survivors with severe COVID-19. (**A**) CE 20:4 of survivors and non-survivors. (**B**) Free cholesterol (FC)/cholesteryl ester (CE) ratio of survivors and non-survivors. * *p* < 0.05.

CE 20:4 (*p* = 0.031) was reduced in serum of non-survivors (Figure 6A). While CE (*p* = 0.190) and FC levels (*p* = 0.170) did not significantly change, the FC/CE ratio of non-survivors was higher in comparison to survivors (*p* = 0.012, Figure 6B).

Viral load was not related to death (*p* = 0.974) but non-survivors had higher antibody titer (*p* = 0.045).

## 4. Discussion

The findings of this study indicate that elevated serum PCSK9 levels in patients with COVID-19 are only modestly associated with disease severity and serum cholesterol levels. Severe COVID-19 patients exhibited elevated FC levels, and the FC/CE ratio was positively correlated with inflammation. Furthermore, the FC/CE ratio was higher in non-survivors, suggesting a potential association between this ratio and the severity and outcome of COVID-19 disease.

Previous research has demonstrated that plasma PCSK9 levels are elevated in septic patients with COVID-19 compared to septic patients without SARS-CoV-2 infection [18]. Current evidence suggests that blood PCSK9 levels are elevated in moderate COVID-19 disease compared to non-infected, healthy controls and are further elevated in severe COVID-19 disease. Serum PCSK9 levels were approximately 2.2-fold higher in moderate cases than in controls and approximately 2.4-fold higher in severe cases than in controls. A similar, approximately 2-fold increase in plasma PCSK9 was reported in COVID-19 patients compared to healthy controls [18]. This shows that SARS-CoV-2 infection induces serum PCSK9, which is only weakly related to disease severity. Accordingly, PCSK9 levels were not correlated with inflammation markers, bacterial and fungal superinfections, or herpes simplex virus reactivation, which are all associated with disease severity and outcome [36,37,38,39,40].

Interestingly, serum PCSK9 correlated with viral titer in moderate but not severe COVID-19. However, whether viral load is related to disease severity is still not clear [41]. This observation suggests that viral load is modestly associated with serum PCSK9 levels, whereas this association is absent in severe cases.

Moreover, serum PCSK9 levels of survivors and non-survivors were similar. Plasma PCSK9 levels did not correlate with survival in hospitalized COVID-19 patients [13] and in septic patients with COVID-19 [18] in accordance with the current findings.

PCSK9 blocking antibodies are used to lower serum cholesterol levels and also improve COVID-19 severity and outcome [7,17]. The beneficial effects of PCSK9 blockage in patients with severe COVID-19 can not be explained by a strong increase in PCSK9 in severe COVID-19 cases because the levels between severe and moderate cases did not greatly differ. In the severe COVID-19 patients, there was a decrease in the serum levels of several CE species. Therefore, a further reduction in serum cholesterol levels by blockage of PCSK9 in SARS-CoV-2 infection may not resemble the protective mechanism of this treatment. PCSK9 blockage reduced circulating IL-6 and was most effective in patients with intense inflammation, indicating that PCSK9 blocking antibodies exert anti-inflammatory effects in SARS-CoV-2 infection [17]. Extracellular PCSK9 enhanced the degradation of angiotensin-converting enzyme 2 (ACE2) used by SARS-CoV-2 to infect the cells [32]. Low levels of PCSK9 may be related to higher ACE2 activity, which exerts anti-inflammatory activities. However, whether PCSK9 antibodies prevent PCSK9-induced degradation of ACE2 needs further study [32,42].

PCSK9 levels are positively associated with serum cholesterol levels [7,43,44], and positive correlations of PCSK9 with CE 18:3 and 20:5 were observed in moderate COVID-19. In severe cases, PCSK9 did not correlate with any of the 15 CE species measured. Patients with PCSK9 loss-of-function variants had lower levels of CE 16:0, 18:1, and 18:3 [45], and the association of PCSK9 with specific CE species remains to be clarified.

Most CE species in human serum are produced by lecithin-cholesterol acyltransferase (LCAT). LCAT mainly forms CE 20:4, 22:5, and 22:6. CE 14:0, 16:0, 18:1, and 18:3 are derived from liver acyl-CoA:cholesterol acyltransferase (ACAT) [46,47,48]. Lower levels of CE 14:0, 14:1, 15:0, and 15:1 in our patients with severe COVID-19 may result from reduced hepatic ACAT activity and lower levels of CE 20:4 from reduced conversion of FC to CE by LCAT. Impaired LCAT activity in sepsis patients has been described before [49]. However, some CE species did not differ between moderate and severe COVID-19 disease, and CE 20:5 was induced in severe cases, indicating that further pathways are involved.

In severe cases of COVID-19, there was a shift from saturated to unsaturated CE species and a decline in CE species with 14-, 15-, and 16-carbon fatty acids. This suggests that the blockade of ACAT activity was more excessive than that of LCAT activity.

In response to infection, cells can produce 25-hydroxycholesterol. This metabolite induces ACAT and plays a role in the depletion of accessible plasma membrane cholesterol, which has anti-microbial effects. This protective role of 25-hydroxycholesterol was lost in ACAT-deficient cells [50], and ACAT downregulation may predispose patients to more severe infectious disease. Lower levels of ACAT-derived CE species indicate lower ACAT activity in severe COVID-19 and suggest that the antimicrobial effect of 25-hydroxycholesterol is impaired.

It is important to note that not all studies have found a link between circulating cholesterol levels and SARS-CoV-2 infection. Cholesterol levels were similar in COVID-19 patients not hospitalized, hospitalized in a normal ward, and in intensive care [51]. Cholesterol and LDL levels were also comparable in COVID-19 patients and healthy controls [52]. Lipidomic profiling of 22 CE species in the serum of 17 severe and 16 moderate COVID-19 cases showed an increase in CE 17:1 and a decline in CE 20:1 but did not imply gross dysregulation of other CE species [53]. In our study cohort, CE species 14:0, 14:1, 15:0, 15:1, 16:0, and 20:4 declined and CE 22:4 and FC were increased in severe compared to moderate COVID-19. CE 17:1 and 20:1 were not analyzed in our study, and information on their regulation in severe COVID-19 cannot be provided.

It has been observed that patients with Gram-positive and Gram-negative bacterial infections also have decreased total cholesterol levels [54]. In our cohort, cholesterol levels did not decline in patients with severe COVID-19 and superinfection with bacteria, fungi, or reactivation of HSV. The effects of bacterial superinfections on specific CE species were moderate and not consistent between moderate and severe cases, thus excluding a close association.

All of the CE species analyzed in our study cohort were negatively correlated with CRP, most of them with procalcitonin and some with IL-6 and ferritin in severe COVID-19. The FC/CE ratio was positively correlated with inflammatory measures in moderate and severe COVID-19. Lower levels of CE 20:4 were modestly related to mortality, and the FC/CE ratio was induced in non-survivors. This shows that inappropriate levels of CE relative to FC are related to disease severity and outcome of patients with COVID-19.

The liver is the most relevant organ for serum PCSK9 and cholesterol levels [7,34]. In patients with COVID-19 and liver cirrhosis, PCSK9 and six CE species were reduced. Studies analyzing the associations of PCSK9 and cholesterol with measures of disease severity and outcome should consider this.

Day of blood collection was not associated with serum PCSK9 levels in our moderate and severe COVID-19 cohort. Notably, in severe COVID-19, CRP and procalcitonin did not correlate with the day of blood collection, showing that this was not associated with this clinically useful marker of inflammation. However, several CE species and FC positively correlated with day of blood sampling. Whether this indicates normalization of cholesterol metabolism during recovery from disease needs further study.

Hypertension, type 2 diabetes, and cardiovascular disease are risk factors for severe COVID-19 disease [6]. For an unknown reason, the prevalence of these comorbidities was similar between our moderate and severe cohorts, and cardiovascular disease had a higher prevalence in our moderate cases. These comorbidities were not associated with altered serum PCSK9 levels, and CE species and FC mostly did not change. Higher levels of some CE species in patients with severe COVID-19 and type 2 diabetes seem to be explained by the increased BMI of diabetic patients, which was found to be positively correlated with CE species levels.

Gender, age, and BMI are further confounding factors of observational studies. PCSK9 levels did not differ between sexes in the cohorts studied herein, and this is in accordance with previous observations [55,56]. Associations of PCSK9 with age have not been consistently reported [55,57,58], and no correlations of serum PCSK9 with age were observed in our patients. In patients with sepsis, plasma PCSK9 was not associated with age [18].

CE 16:0, CE 18:2, CE 20:4, and CE 22:6 were negatively correlated with age in the moderate COVID-19 patients, whereas associations with age did not exist in the severe cohort. This suggests that correlations of serum CE levels with age depend on parameters of the underlying health condition. Both sexes had similar levels of CE species and FC, showing that sex is not a confounding factor for the analysis of CE and FC in severe illness.

Adiposity is a risk factor for severe COVID-19 [6], and there were more obese patients in the severe COVID-19 cohort compared to patients with moderate disease. Higher serum PCSK9 levels in obese subjects in comparison to normal-weight controls were reported [8]. The lack of an association of serum PCSK9 with adiposity in SARS-CoV-2 infection indicates no major role of BMI for serum PCSK9 levels in COVID-19.

This is different for the CE species levels. In the moderate COVID-19 group, CE 20:3, and in the severe cohort, 10 of the 15 analyzed CE species were positively related to BMI. A correlation was observed between overweight and mild obesity and a reduced mortality rate among sepsis patients. However, these conditions were also linked to an elevated risk of developing severe forms of COVID-19 [6,59]. A higher BMI was associated with elevated levels of CEs, but whether this indicates a protective effect of increased BMI that is offset by the adverse consequences of obesity, such as low-grade inflammation, can not be resolved in our observational study.

It is noteworthy that patients with severe COVID-19 exhibited a higher SARS-CoV-2 antibody titer in comparison to patients with moderate disease [60], as observed in our study. Antibody levels were not correlated with PCSK9, and the negative correlation of CE species with antibody levels in patients with severe COVID-19 seems to be related to disease severity.

This study has limitations. Blood was obtained during the hospital stay and not always early after hospital admission. We did not analyze serum PCSK9 levels during therapy and can not provide information on whether levels normalize during recovery. Commonly used drugs such as statins were not documented. The total serum cholesterol levels of our cohort was 3.1 (1.6–7.8) mmol/L, showing that most of our patients had quite normal levels [61]. CE and FC levels of healthy controls were not measured in this study. Moreover, this observational study cannot provide information about the underlying processes contributing to higher circulating PCSK9, altered CE profile, and FC levels in COVID-19.

## 5. Conclusions

This study offers insights into cholesterol metabolism in COVID-19 and proposes the FC/CE ratio as a biomarker for predicting disease severity and mortality in COVID-19.

## Figures and Tables

**Table 1:** Data of the patients with COVID-19. Data are shown as median values, and the minimum and maximum values are given in parentheses. In instances where data were unavailable for all patients, the numbers in capital letters indicate the number of patients for whom these data had been documented. (alkaline phosphatase: AP, arbitrary unit: AU, body mass index: BMI, interleukin: IL, lactate dehydrogenase; LDH).

Parameter	Moderate COVID-19	Severe COVID-19
Males/Females	22/18	42/18
Age (years)	60 (22–83)	57 (31–83)
BMI (kg/m^2^)	26 (18–43) ^23^	29 (19–67) ^56^ **
C-reactive protein (mg/L)	25 (0–218)	74 (1–367) **
Procalcitonin (ng/mL)	0.09 (0–25) ^31^	0.24 (0–25) **
LDH (U/L)	224 (127–929)	378 (162–1534) ***
AP (U/L)	92 (38–372) ^30^	99 (37–743)
Ferritin (ng/mL)	601 (68–4237) ^27^	1088 (77–21,976) *
IL-6 (pg/mL)	30 (4–265) ^22^	36 (3–1175)
Lactate (mg/dL)	10 (4–20) ^6^	10 (4–25)
Viral Load (cp/mL)	4600 (48–19 × 10^6^) ^39^	14 × 10^3^ (95–520 × 10^6^) ^49^
Antibody (AU/mL)	60 (14–848) ^7^	661 (17–1939) ^47^ *
Hypertension	5	2
Diabetes	11	12
Cardiovascular Disease	8	2 **

* *p* < 0.05, ** *p* < 0.01, and *** *p* < 0.001.

**Table 2:** Spearman correlation coefficients (r) for the correlation of serum cholesteryl ester (CE) species, free cholesterol (FC), and the FC/CE ratio with C-reactive protein, procalcitonin, IL-6, and ferritin.

	C-Reactive Protein		Procalcitonin		IL-6		Ferritin	
CE	Moderate	Severe	Moderate	Severe	Moderate	Severe	Moderate	Severe
14:0	−0.276	−0.564 ***	−0.033	−0.474 ***	−0.050	−0.333 *	−0.207	−0.441 ***
14:1	−0.373 *	−0.291 *	−0.102	−0.176	−0.288	−0.311 *	−0.134	−0.085
15:0	−0.224	−0.481 ***	−0.066	−0.438 ***	−0.029	−0.274 *	−0.191	−0.378 **
15:1	−0.078	−0.353 **	0.017	−0.174	−0.237	−0.258 *	−0.124	−0.115
16:0	−0.284	−0.529 ***	−0.305	−0.569 ***	−0.083	−0.341 **	−0.248	−0.499 ***
16:1	−0.223	−0.470 ***	−0.153	−0.387 **	−0.017	−0.322 *	−0.159	−0.339 **
18:1	−0.254	−0.529 ***	−0.192	−0.460 ***	0.104	−0.330 *	−0.024	−0.318 *
18:2	−0.266	−0.421 **	−0.259	−0.515 ***	−0.093	−0.291 *	−0.322	−0.342 **
18:3	−0.051	−0.489 ***	−0.136	−0.494 ***	−0.206	−0.243	−0.234	−0.339 **
20:3	−0.240	−0.281 *	−0.011	−0.348 **	−0.364	−0.046	−0.353	−0.329 *
20:4	−0.177	−0.494 ***	−0.494 **	−0.586 ***	−0.093	−0.341 **	−0.276	−0.491 ***
20:5	−0.034	−0.338 **	−0.247	−0.365 **	0.081	−0.082	−0.035	−0.203
22:4	−0.038	−0.372 **	−0.261	−0.345 **	0.011	−0.239	0.027	−0.221
22:5	−0.218	−0.324 *	−0.238	−0.315 *	0.015	−0.147	−0.046	−0.228
22:6	−0.126	−0.414 **	−0.440 *	−0.517 ***	−0.093	−0.236	−0.205	−0.364 **
FC	0.218	−0.040	0.263	0.079	0.438	0.056	0.197	−0.052
FC/CE	0.635 ***	0.608 ***	0.475 *	0.757 ***	0.585 **	0.460 ***	0.428 *	0.440 ***

* *p* < 0.05, ** *p* < 0.01, *** *p* < 0.001.

**Table 3:** Correlation of CE species with body mass index in moderate and severe COVID-19.

CE	Moderate	Severe
14:0	0.009	0.407 **
14:1	−0.007	0.328 *
15:0	−0.018	0.353 *
15:1	0.193	0.298 *
16:0	0.067	0.436 **
16:1	0.049	0.519 ***
18:1	−0.070	0.425 **
18:2	0.146	0.185
18:3	0.191	0.305 *
20:3	0.561 *	0.257
20:4	−0.035	0.484 ***
20:5	−0.016	0.090
22:4	0.226	0.230
22:5	−0.069	0.160
22:6	−0.082	0.313 *
FC	−0.302	0.076
FC/CE	−0.442	−0.335 *

* *p* < 0.05, ** *p* < 0.01, *** *p* < 0.001.

## Data Availability

The data that support the findings of this study are available from the corresponding author (C.B.) upon reasonable request.

## Supplementary Materials

The following supporting information can be downloaded at https://www.mdpi.com/article/10.3390/idr16040045/s1: Figure S1: PCSK9 prevents LDL receptor recycling to the cell surface, redirecting the receptor to lysosomes for degradation. This results in higher circulating total and LDL cholesterol levels.

## References

[1] Khot W.Y., Nadkar M.Y. (2020). The 2019 Novel Coronavirus Outbreak-A Global Threat. J. Assoc. Physicians India.

[2] Diarimalala R.O., Wei Y., Hu D., Hu K. (2023). Inflammasomes during SARS-CoV-2 infection and development of their corresponding inhibitors. Front. Cell Infect. Microbiol..

[3] Shah M.D., Sumeh A.S., Sheraz M., Kavitha M.S., Venmathi Maran B.A., Rodrigues K.F. (2021). A mini-review on the impact of COVID 19 on vital organs. Biomed. Pharmacother..

[4] Abbott M., Li Y., Brochard L., Zhang H. (2023). Precision Medicine Using Simultaneous Monitoring and Assessment with Imaging and Biomarkers to Manage Mechanical Ventilation in ARDS. Intensive Care Res..

[5] Zsichla L., Muller V. (2023). Risk Factors of Severe COVID-19: A Review of Host, Viral and Environmental Factors. Viruses.

[6] Grewal T., Buechler C. (2023). Adipokines as Diagnostic and Prognostic Markers for the Severity of COVID-19. Biomedicines.

[7] Grewal T., Buechler C. (2022). Emerging Insights on the Diverse Roles of Proprotein Convertase Subtilisin/Kexin Type 9 (PCSK9) in Chronic Liver Diseases: Cholesterol Metabolism and Beyond. Int. J. Mol. Sci..

[8] Filippatos T.D., Liberopoulos E., Georgoula M., Tellis C.C., Tselepis A.D., Elisaf M. (2017). Effects of increased body weight and short-term weight loss on serum PCSK9 levels—A prospective pilot study. Arch. Med. Sci. Atheroscler. Dis..

[9] Barlage S., Gnewuch C., Liebisch G., Wolf Z., Audebert F.X., Gluck T., Frohlich D., Kramer B.K., Rothe G., Schmitz G. (2009). Changes in HDL-associated apolipoproteins relate to mortality in human sepsis and correlate to monocyte and platelet activation. Intensive Care Med..

[10] Deniz O., Gumus S., Yaman H., Ciftci F., Ors F., Cakir E., Tozkoparan E., Bilgic H., Ekiz K. (2007). Serum total cholesterol, HDL-C and LDL-C concentrations significantly correlate with the radiological extent of disease and the degree of smear positivity in patients with pulmonary tuberculosis. Clin. Biochem..

[11] Hofmaenner D.A., Kleyman A., Press A., Bauer M., Singer M. (2022). The Many Roles of Cholesterol in Sepsis: A Review. Am. J. Respir. Crit. Care Med..

[12] Patel P.N., Shah R.Y., Ferguson J.F., Reilly M.P. (2015). Human experimental endotoxemia in modeling the pathophysiology, genomics, and therapeutics of innate immunity in complex cardiometabolic diseases. Arterioscler. Thromb. Vasc. Biol..

[13] Momtazi-Borojeni A.A., Sabouri-Rad S., Gotto A.M., Pirro M., Banach M., Awan Z., Barreto G.E., Sahebkar A. (2019). PCSK9 and inflammation: A review of experimental and clinical evidence. Eur. Heart J. Cardiovasc. Pharmacother..

[14] Walley K.R., Thain K.R., Russell J.A., Reilly M.P., Meyer N.J., Ferguson J.F., Christie J.D., Nakada T.A., Fjell C.D., Thair S.A. (2014). PCSK9 is a critical regulator of the innate immune response and septic shock outcome. Sci. Transl. Med..

[15] Boyd J.H., Fjell C.D., Russell J.A., Sirounis D., Cirstea M.S., Walley K.R. (2016). Increased Plasma PCSK9 Levels Are Associated with Reduced Endotoxin Clearance and the Development of Acute Organ Failures during Sepsis. J. Innate Immun..

[16] Zhou Z., Zhang W., Burgner D., Tonkin A., Zhu C., Sun C., Magnussen C.G., Ernst M.E., Breslin M., Nicholls S.J. (2023). The association between PCSK9 inhibitor use and sepsis-A systematic review and meta-analysis of 20 double-blind, randomized, placebo-controlled trials. Am. J. Med..

[17] Navarese E.P., Podhajski P., Gurbel P.A., Grzelakowska K., Ruscio E., Tantry U., Magielski P., Kubica A., Niezgoda P., Adamski P. (2023). PCSK9 Inhibition During the Inflammatory Stage of SARS-CoV-2 Infection. J. Am. Coll. Cardiol..

[18] Mester P., Amend P., Schmid S., Muller M., Buechler C., Pavel V. (2023). Plasma Proprotein Convertase Subtilisin/Kexin Type 9 (PCSK9) as a Possible Biomarker for Severe COVID-19. Viruses.

[19] Ruscica M., Macchi C., Iodice S., Tersalvi G., Rota I., Ghidini S., Terranova L., Valenti L., Amati F., Aliberti S. (2021). Prognostic parameters of in-hospital mortality in COVID-19 patients-An Italian experience. Eur. J. Clin. Investig..

[20] Bone R.C. (1995). Sepsis, sepsis syndrome, and the systemic inflammatory response syndrome (SIRS). Gulliver in Laputa. JAMA.

[21] Singer M., Deutschman C.S., Seymour C.W., Shankar-Hari M., Annane D., Bauer M., Bellomo R., Bernard G.R., Chiche J.D., Coopersmith C.M. (2016). The Third International Consensus Definitions for Sepsis and Septic Shock (Sepsis-3). JAMA.

[22] https://www.covid19treatmentguidelines.nih.gov/overview/clinical-spectrum/.

[23] Evans L., Rhodes A., Alhazzani W., Antonelli M., Coopersmith C.M., French C., Machado F.R., McIntyre L., Ostermann M., Prescott H.C. (2021). Surviving Sepsis Campaign: International Guidelines for Management of Sepsis and Septic Shock 2021. Crit. Care Med..

[24] Karakike E., Giamarellos-Bourboulis E.J., Kyprianou M., Fleischmann-Struzek C., Pletz M.W., Netea M.G., Reinhart K., Kyriazopoulou E. (2021). Coronavirus Disease 2019 as Cause of Viral Sepsis: A Systematic Review and Meta-Analysis. Crit. Care Med..

[25] Bligh E.G., Dyer W.J. (1959). A rapid method of total lipid extraction and purification. Can. J. Biochem. Physiol..

[26] Horing M., Ejsing C.S., Krautbauer S., Ertl V.M., Burkhardt R., Liebisch G. (2021). Accurate quantification of lipid species affected by isobaric overlap in Fourier-transform mass spectrometry. J. Lipid Res..

[27] Horing M., Ekroos K., Baker P.R.S., Connell L., Stadler S.C., Burkhardt R., Liebisch G. (2020). Correction of Isobaric Overlap Resulting from Sodiated Ions in Lipidomics. Anal. Chem..

[28] Horing M., Ejsing C.S., Hermansson M., Liebisch G. (2019). Quantification of Cholesterol and Cholesteryl Ester by Direct Flow Injection High-Resolution Fourier Transform Mass Spectrometry Utilizing Species-Specific Response Factors. Anal. Chem..

[29] Hitzenbichler F., Bauernfeind S., Salzberger B., Schmidt B., Wenzel J.J. (2021). Comparison of Throat Washings, Nasopharyngeal Swabs and Oropharyngeal Swabs for Detection of SARS-CoV-2. Viruses.

[30] Peterhoff D., Gluck V., Vogel M., Schuster P., Schutz A., Neubert P., Albert V., Frisch S., Kiessling M., Pervan P. (2021). A highly specific and sensitive serological assay detects SARS-CoV-2 antibody levels in COVID-19 patients that correlate with neutralization. Infection.

[31] Idelevich E.A., Reischl U., Becker K. (2018). New Microbiological Techniques in the Diagnosis of Bloodstream Infections. Dtsch. Arztebl. Int..

[32] Essalmani R., Andreo U., Evagelidis A., Le Devehat M., Pereira Ramos O.H., Fruchart Gaillard C., Susan-Resiga D., Cohen E.A., Seidah N.G. (2023). SKI-1/S1P Facilitates SARS-CoV-2 Spike Induced Cell-to-Cell Fusion via Activation of SREBP-2 and Metalloproteases, Whereas PCSK9 Enhances the Degradation of ACE2. Viruses.

[33] Katz J., Yue S., Xue W. (2022). Herpes simplex and herpes zoster viruses in COVID-19 patients. Ir. J. Med. Sci..

[34] Buechler C., Aslanidis C. (2020). Role of lipids in pathophysiology, diagnosis and therapy of hepatocellular carcinoma. Biochim. Biophys. Acta Mol. Cell Biol. Lipids.

[35] Hofmaenner D.A., Arina P., Kleyman A., Page Black L., Salomao R., Tanaka S., Guirgis F.W., Arulkumaran N., Singer M. (2023). Association Between Hypocholesterolemia and Mortality in Critically Ill Patients With Sepsis: A Systematic Review and Meta-Analysis. Crit. Care Explor..

[36] Atallah N.J., Warren H.M., Roberts M.B., Elshaboury R.H., Bidell M.R., Gandhi R.G., Adamsick M., Ibrahim M.K., Sood R., Bou Zein Eddine S. (2022). Baseline procalcitonin as a predictor of bacterial infection and clinical outcomes in COVID-19: A case-control study. PLoS ONE.

[37] Garcia-Vidal C., Sanjuan G., Moreno-Garcia E., Puerta-Alcalde P., Garcia-Pouton N., Chumbita M., Fernandez-Pittol M., Pitart C., Inciarte A., Bodro M. (2021). Incidence of co-infections and superinfections in hospitalized patients with COVID-19: A retrospective cohort study. Clin. Microbiol. Infect..

[38] Musuuza J.S., Watson L., Parmasad V., Putman-Buehler N., Christensen L., Safdar N. (2021). Prevalence and outcomes of co-infection and superinfection with SARS-CoV-2 and other pathogens: A systematic review and meta-analysis. PLoS ONE.

[39] Chiesa A.F., Pallanza M., Martinetti G., Lanzi F., Previsdomini M., Pagnamenta A., Elzi L. (2022). Herpes simplex virus reactivation in patients with COVID-19 and acute respiratory distress syndrome: A prospective cohort study. Antivir. Ther..

[40] Giacobbe D.R., Di Bella S., Dettori S., Brucci G., Zerbato V., Pol R., Segat L., D’Agaro P., Roman-Pognuz E., Friso F. (2022). Reactivation of Herpes Simplex Virus Type 1 (HSV-1) Detected on Bronchoalveolar Lavage Fluid (BALF) Samples in Critically Ill COVID-19 Patients Undergoing Invasive Mechanical Ventilation: Preliminary Results from Two Italian Centers. Microorganisms.

[41] Dadras O., Afsahi A.M., Pashaei Z., Mojdeganlou H., Karimi A., Habibi P., Barzegary A., Fakhfouri A., Mirzapour P., Janfaza N. (2022). The relationship between COVID-19 viral load and disease severity: A systematic review. Immun. Inflamm. Dis..

[42] Grewal T., Nguyen M.K.L., Buechler C. (2024). Cholesterol and COVID-19-therapeutic opportunities at the host/virus interface during cell entry. Life Sci. Alliance.

[43] Nozue T. (2017). Lipid Lowering Therapy and Circulating PCSK9 Concentration. J. Atheroscler. Thromb..

[44] Sundararaman S.S., Doring Y., van der Vorst E.P.C. (2021). PCSK9: A Multi-Faceted Protein That Is Involved in Cardiovascular Biology. Biomedicines.

[45] Janis M.T., Tarasov K., Ta H.X., Suoniemi M., Ekroos K., Hurme R., Lehtimaki T., Paiva H., Kleber M.E., Marz W. (2013). Beyond LDL-C lowering: Distinct molecular sphingolipids are good indicators of proprotein convertase subtilisin/kexin type 9 (PCSK9) deficiency. Atherosclerosis.

[46] Glomset J.A. (1979). Lecithin: Cholesterol acyltransferase. An exercise in comparative biology. Prog. Biochem. Pharmacol..

[47] Pramfalk C., Eriksson M., Parini P. (2012). Cholesteryl esters and ACAT. Eur. J. Lipid Sci. Technol..

[48] Subbaiah P.V., Jiang X.C., Belikova N.A., Aizezi B., Huang Z.H., Reardon C.A. (2012). Regulation of plasma cholesterol esterification by sphingomyelin: Effect of physiological variations of plasma sphingomyelin on lecithin-cholesterol acyltransferase activity. Biochim. Biophys. Acta.

[49] Reisinger A.C., Schuller M., Sourij H., Stadler J.T., Hackl G., Eller P., Marsche G. (2021). Impact of Sepsis on High-Density Lipoprotein Metabolism. Front. Cell Dev. Biol..

[50] Heisler D.B., Johnson K.A., Ma D.H., Ohlson M.B., Zhang L., Tran M., Corley C.D., Abrams M.E., McDonald J.G., Schoggins J.W. (2023). A concerted mechanism involving ACAT and SREBPs by which oxysterols deplete accessible cholesterol to restrict microbial infection. eLife.

[51] Lavis P., Morra S., Orte Cano C., Albayrak N., Corbiere V., Olislagers V., Dauby N., Del Marmol V., Marchant A., Decaestecker C. (2022). Chemerin plasma levels are increased in COVID-19 patients and are an independent risk factor of mortality. Front. Immunol..

[52] Kukla M., Menzyk T., Dembinski M., Winiarski M., Garlicki A., Bociaga-Jasik M., Skonieczna M., Hudy D., Maziarz B., Kusnierz-Cabala B. (2021). Anti-inflammatory adipokines: Chemerin, vaspin, omentin concentrations and SARS-CoV-2 outcomes. Sci. Rep..

[53] Caterino M., Gelzo M., Sol S., Fedele R., Annunziata A., Calabrese C., Fiorentino G., D’Abbraccio M., Dell’Isola C., Fusco F.M. (2021). Dysregulation of lipid metabolism and pathological inflammation in patients with COVID-19. Sci. Rep..

[54] Feingold K.R., Grunfeld C., Feingold K.R., Anawalt B., Blackman M.R., Boyce A., Chrousos G., Corpas E., de Herder W.W., Dhatariya K., Dungan K., Hofland J. (2000). The Effect of Inflammation and Infection on Lipids and Lipoproteins. Endotext.

[55] Grimm J., Peschel G., Muller M., Schacherer D., Wiest R., Weigand K., Buechler C. (2021). Rapid Decline of Serum Proprotein Convertase Subtilisin/Kexin 9 (PCSK9) in Non-Cirrhotic Patients with Chronic Hepatitis C Infection Receiving Direct-Acting Antiviral Therapy. J. Clin. Med..

[56] Marinelli C., Zingone F., Lupo M.G., Marin R., D’Inca R., Gubbiotti A., Massimi D., Casadei C., Barberio B., Ferri N. (2022). Serum Levels of PCSK9 Are Increased in Patients With Active Ulcerative Colitis Representing a Potential Biomarker of Disease Activity: A Cross-sectional Study. J. Clin. Gastroenterol..

[57] Ghosh M., Galman C., Rudling M., Angelin B. (2015). Influence of physiological changes in endogenous estrogen on circulating PCSK9 and LDL cholesterol. J. Lipid Res..

[58] Rannikko J., Jacome Sanz D., Ortutay Z., Seiskari T., Aittoniemi J., Huttunen R., Syrjanen J., Pesu M. (2019). Reduced plasma PCSK9 response in patients with bacteraemia is associated with mortality. J. Intern. Med..

[59] Karampela I., Christodoulatos G.S., Dalamaga M. (2019). The Role of Adipose Tissue and Adipokines in Sepsis: Inflammatory and Metabolic Considerations, and the Obesity Paradox. Curr. Obes. Rep..

[60] Guo Y., Li T., Xia X., Su B., Li H., Feng Y., Han J., Wang X., Jia L., Bao Z. (2021). Different Profiles of Antibodies and Cytokines Were Found Between Severe and Moderate COVID-19 Patients. Front. Immunol..

[61] Schucker B., Wittes J.T., Santanello N.C., Weber S.J., McGoldrick D., Donato K., Levy A., Rifkind B.M. (1991). Change in cholesterol awareness and action. Results from national physician and public surveys. Arch. Intern. Med..

