# Supplementary material for: Proprotein Convertase Subtilisin/Kexin Type 9 Induction in COVID-19 Is Poorly Associated with Disease Severity and Cholesterol Levels
Source: Infect Dis Rep. 2024 Jul 17;16(4):593–607. doi: 10.3390/idr16040045 (PMC11270413; doi:10.3390/idr16040045)
Supplement: Supplementary file 1 [file idr-16-00045-s001.zip › idr-3051489-supplementary.pdf]

Proprotein convertase subtilisin/kexin type 9 induction in COVID-19 is poorly associated with disease severity and cholesterol levels

Patricia Mester [1], Pablo Amend [1], Stephan Schmid [1], Jürgen J Wenzel [2], Marcus Höring [3], Gerhard Liebisch [3], Sabrina Krautbauer [3], Martina Müller [1], Christa Buechler [1,*,§] and Vlad Pavel [1,§]

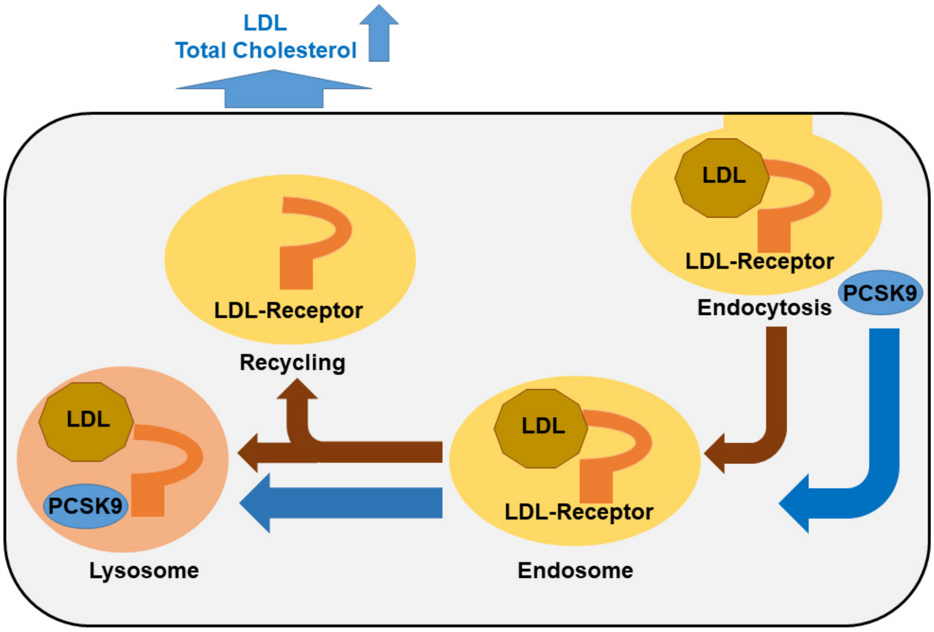

Figure S1. PCSK9 prevents LDL receptor recycling to the cell surface, redirecting the receptor to lysosomes for degradation. This results in higher circulating total cholesterol and LDL levels.

.
